# A Feasible Path to Reductions in Racial and Ethnic Disparities in Lung Cancer Screening?

**DOI:** 10.1093/jncics/pkac034

**Published:** 2022-05-10

**Authors:** Debra P Ritzwoller

**Affiliations:** Institute for Health Research, Kaiser Permanente Colorado, Aurora, CO, USA

Racial and ethnic disparities exist and persist, both in the uptake of lung cancer screening (LCS) and in lung cancer mortality rates (1-3). In 2021, the United States Preventive Services Task Force (USPSTF) updated their recommendations for LCS, lowering the age from 55 to 50 years and smoking history from 30 to 20 pack-years (4). This update was informed by analyses from the Cancer Intervention and Surveillance Modeling Network Lung Cancer Working Group, which found that these changes could partially address disparities in screening eligibility and, therefore, lung cancer incidence and mortality among racial and ethnic minorities (5). Recent studies evaluating the potential population impact of LCS under the 2021 USPSTF criteria using survey and/or electronic health record (EHR) data found improvements in eligibility for individuals in racial and ethnic minority groups (6-8). Despite these potential improvements, LCS inequities within racial and ethnic minority groups may perpetuate without enhanced outreach and without the addition of tailored eligibility criteria that address risk factors for lung cancer other than smoking history and age.

The 2012 modification of the model from the Prostate, Lung, Colorectal, and Ovarian Cancer Screening Trial (PLCOm2012) is a risk-based prediction model that has been shown to reduce racial and ethnic LCS eligibility disparities relative to the 2013 USPSTF criteria and, more recently, relative to the 2021 USPSTF criteria (9-11). In addition to smoking status and intensity, the PLCOm2012 employs a comprehensive set of patient-level risk factors, including chronic obstructive pulmonary disease, education level, and family and personal history of cancer. Whereas eligibility assessments comparing both 2013 USPSTF criteria relative to PLCOm2012 have been well studied, less is known regarding the efficiency of these models to diagnose lung cancer in large cohorts of racially and ethnically diverse, ever-smoker patients.

In this issue of the Journal, Aredo and colleagues (12) take advantage of the Multiethnic Cohort Study (MEC) to assess the advantages of the 2021 updated USPSTF criteria relative to a risk-based approach. The MEC–Surveillance, Epidemiology, and End Results registry linkages along with the density of understudied minorities provide a unique and optimal resource for this study of the impact of alternative screening guidelines on racial and ethnic disparities in LCS (13). Given the importance of addressing inequities in LCS, and in the diagnosis of lung cancer, the topic addressed in this article is both important and timely. Applying both the 2013 and 2021 USPSTF LCS eligibility criteria and the PLCOm2012 risk-based criteria to a MEC subpopulation of individuals with noted smoking histories, the authors demonstrate that the 2021 USPSTF21 criteria reduces disparities relative to the USPSTF13 criteria but that risk-based screening may achieve a greater screening sensitivity in some racial and ethnic groups. Specifically, the conundrum the authors demonstrate is that screening disparity decreased from 11.2% to 5.1% for African Americans using the PLCOm2012 risk-based model relative 2021 USPSTF, but screening disparities increased from 9.6% to 12.8% for Japanese Americans and from 12.4% to 28.6% for Latinos. The MEC is an older cohort of diverse individuals aged between 45 and 75 years during the 1993-1996 study recruitment period. Environmental and dietary exposures and smoking patterns have changed over the past 30 years, suggesting possible selection effects among racial and ethnic groups that could differ from the original PLCOm2012 development and validation cohorts (14).

However, this provocative finding of racial disparity amelioration via use of the PLCOm2012 risk-based criteria is echoed, if not amplified, in a similar analysis by Pu et al. (15). In this study that used a Detroit area cohort of 912 White and African America patients diagnosed with lung cancer, the authors find the White vs African America 10-percentage-point sensitivity disparity noted under the 2013 USPSTF criteria (White patients [52%], African American patients [42%], *P* = .007) was completely eliminated using either 2021 USPSTF criteria (White patients [65%] vs African American patients [63%], *P* = .64) or the PLCOm2012 criteria (White patients [68%] vs African American patients [67%], *P* = .73).

The findings reported by Aredo and colleagues (12) add to the growing body of evidence demonstrating the superiority of the 2021 USPSTF criteria over the 2013 USPSTF and that risk-based screening consistent with PLCOm2012 criteria may be superior to both the 2013 and the 2021 USPSTF guidelines with respect to reducing racial disparities in lung cancer. But the path to improving disparities within and across all historically minoritized populations may require much more of a lift than just optimizing the risk-based prediction models. Specifically, the ability of providers and health-care systems to accurately ascertain lung cancer–related risk factors at the individual and population levels is sorely lacking. Efforts to enhance the feasibility of collecting population measures of lung cancer risk factors is key, especially given that providers and health-care systems currently have difficulties collecting comprehensive measures of smoking history and smoking intensity. A recent study that employed EHR data derived from 5 diverse community-based health-care systems found 54% of insured current or former smokers with access to primary care providers lacked information on pack-years and cessation dates (8). Moreover, the capture of education level and individual or family history of cancer, key parameters in the PLCOm2012, is not well integrated into our current clinical care processes (16-19).

In this study, the authors used rigorous imputation strategies to address missing key risk factors and temporal gaps between the assessment of a patient's smoking behavior at enrollment vs at the time of lung cancer diagnosis. How does the absence of key lung cancer risk factors translate in busy community practices? Patient-level lung cancer risk factors may be captured at 1 point in time, in 1 EHR, but they may not be captured downstream or visible to the patient’s current provider if the patient changes health-care systems or loses health-care coverage (20). Currently, a variety of web-based lung cancer risk calculators provide real-time risk prediction and screening recommendations via the capture and input key patient-level lung cancer risk factors (21). These tools may enhance the patient-provider engagement and shared decision-making discussions related to the harms and benefits of LCS, but most are not integrated with EHRs, thus limiting their use for ascertaining populations or health-care system cohorts of LCS eligible patients.

LCS participation in community settings is dismal compared with breast, colorectal, or cervical cancer screening participation (20,22). Relevant to this differential in participation rates is the fact that LCS is the only cancer screening modality covered under the Patient Protection and Affordable Care Act and Centers for Medicare and Medicaid Services payment rules that have eligibility requirements beyond age and sex. Although most commercially available EHRs include modules that allow for the capture of structured or semistructured smoking status variables, underuse is common, thus lessening opportunities to employ EHR-embedded alerts or notifications signaling potential patient eligibility for LCS (16,23).

Implementation of either the 2021 USPSTF criteria or risk-based LCS screening criteria is likely to result in statistical and clinical efficiencies relative to the 2013 USPSTF criteria in identifying substantially more lung cancers and, most important, in reducing racial and ethnic disparities in lung cancer. However, the current path to improvement in LCS participation, and ultimately in the reduction in racial and ethnic disparities, is dependent on providers who are already overwhelmed and may have little incentive, time, or support to establish the infrastructure necessary to perform systematic risk assessment. But the path forward is possible if quality measures and financial incentives are provided at the clinician and health-care-system levels that target enhanced risk factor assessment for all individuals with a smoking history. Evidence provided by this study provides the motivation for the development and implementation of needed intervention strategies to improve the technical and patient/provider communication infrastructure needed to optimize lung cancer risk–factor ascertainment.

## Funding

Not applicable.

## Notes

**Role of the funder:** Not applicable.

**Disclosures:** The author receives support by grants from the National Cancer Institute of the National Institutes of Health under the Award Number UM1CA221939 and R01CA260889.

**Author contributions:** Writing-original draft, writing-review & editing: DPR.

**Disclaimer: **The author alone is solely the responsible for the views expressed in this article and they do not represent the official views, decisions, or policy of the National Institutes of Health.

## Data Availability

No new data were generated or analyzed for this editorial.
